# Human Hepatocyte 4-Acetoxy-*N*,*N*-Diisopropyltryptamine Metabolite Profiling by Reversed-Phase Liquid Chromatography Coupled with High-Resolution Tandem Mass Spectrometry

**DOI:** 10.3390/metabo12080705

**Published:** 2022-07-29

**Authors:** Sara Malaca, Marilyn A. Huestis, Leonardo Lattanzio, Luigi T. Marsella, Adriano Tagliabracci, Jeremy Carlier, Francesco P. Busardò

**Affiliations:** 1 Unit of Forensic Toxicology, Section of Legal Medicine, Department of Excellence of Biomedical Sciences and Public Health, Marche Polytechnic University, Via Tronto 10/a, 60126 Ancona, Italy; smalaca@hotmail.com (S.M.); a.tagliabracci@staff.univpm.it (A.T.); fra.busardo@libero.it (F.P.B.); 2 Institute of Emerging Health Professions, Thomas Jefferson University, Philadelphia, PA 19107, USA; marilyn.huestis@gmail.com; 3 Forensic Medicine, Department of Biomedicine and Prevention, University of Rome Tor Vergata, 00133 Rome, Italy; leonardolattanzio1@gmail.com (L.L.); marsella.luigi@gmail.com (L.T.M.)

**Keywords:** tryptamine, 4-AcO-DiPT, in silico prediction, hepatocyte metabolism, liquid chromatography–high-resolution tandem mass spectrometry (LC-HRMS/MS), data mining

## Abstract

Tryptamine intoxications and fatalities are increasing, although these novel psychoactive substances (NPS) are not controlled in most countries. There are few data on the metabolic pathways and enzymes involved in tryptamine biotransformation. 4-acetoxy-*N*,*N*-diisopropyltryptamine (4-AcO-DiPT) is a synthetic tryptamine related to 4-hydroxy-*N*,*N*-diisopropyltryptamine (4-OH-DiPT), 4-acetyloxy-*N*,*N*-dipropyltryptamine (4-AcO-DPT), and 4-acetoxy-*N*,*N*-dimethyltryptamine (4-AcO-DMT). The aim of this study was to determine the best 4-AcO-DiPT metabolites to identify 4-AcO-DiPT consumption through human hepatocyte metabolism and high-resolution mass spectrometry. 4-AcO-DiPT metabolites were predicted in silico with GLORYx freeware to assist in metabolite identification. 4-AcO-DiPT was incubated with 10-donor-pooled human hepatocytes and sample analysis was performed with reversed-phase liquid chromatography coupled with high-resolution tandem mass spectrometry (LC-HRMS/MS) in positive- and negative-ion modes. Software-assisted LC-HRMS/MS raw data mining was performed. A total of 47 phase I and II metabolites were predicted, and six metabolites were identified after 3 h incubation following ester hydrolysis, *O*-glucuronidation, *O*-sulfation, *N*-oxidation, and *N*-dealkylation. All second-generation metabolites were derived from the only first-generation metabolite detected after ester hydrolysis (4-OH-DiPT). The metabolite with the second-most-intense signal was 4-OH-iPT-sulfate followed by 4-OH-DiPT-glucuronide, indicating that glucuronidation and sulfation are common in this tryptamine’s metabolic pathway. 4-OH-DiPT, 4-OH-iPT, and 4-OH-DiPT-*N*-oxide are suggested as optimal biomarkers to identify 4-AcO-DiPT consumption.

## 1. Introduction

Tryptamines are a class of new psychoactive substances (NPS) that share a core structure with the neurotransmitter serotonin, or 5-hydroxytryptamine (5-HT). Tryptamine’s psychedelic effects are produced through agonism of the 5-HT_2A_ and 5-HT_2C_ receptors, with additional contribution from other monoamine transporters [1,2,3,4,5,6,7,8,9,10]. Diverse tryptamine structures have different receptor affinities and resultant effects. 4-AcO-DiPT is a synthetic tryptamine related to 4-hydroxy-*N*,*N*-diisopropyltryptamine (4-OH-DiPT), 4-acetyloxy-*N*,*N*-dipropyltryptamine (4-AcO-DPT), and 4-acetoxy-*N*,*N*-dimethyltryptamine (4-AcO-DMT), which currently are not controlled in many countries. 4-AcO-DiPT was identified as early as July 2004, with charges against vendors selling the drug to the public; however, there were no convictions and there was no determination of the legal status of 4-AcO-DiPT [11]. Although the current use of psychedelics is low, tryptamine intake is increasing [1,10] despite limited data on prevalence and patterns of intake. According to psychonauts’ experiences, as reported on the Erowid website, 4-AcO-DiPT is available online and typically administrated orally, with effects similar to those of hallucinogenic mushrooms and 2-(4-bromo-2,5-dimethoxyphenyl)-ethanamine (2C-B) [12]. 4-AcO-DiPT is a white powder available in freebase and HCl salt forms. Both are orally active, with the lower molecular weight freebase being about 10% more potent than the same weight of the HCl salt. 4-AcO-DiPT and 4-OH-DiPT have similar effects if taken orally, though dosage might be different. Oral 4-AcO-DiPT doses start at 3–5 mg; 5–15 mg is considered a “light” dose, while higher doses range between 25 and 40 mg. Users self-report 15–30 mg doses, with onset of effects in 20–60 min, depending on dosage form and stomach contents, and duration of 2–4 h. Its effects, which can appear within 1–4 h, are dose-dependent and include hallucination, dissociation, confusion, and flashbacks, as reported on Erowid by a 4-AcO-DPT user [12]. Over the last decade, there were few intoxication cases and even fewer fatalities due to tryptamines reported in the European Union and North America, although this is likely an underestimation due to missed detections. There are few data on metabolic pathways and specific enzymes involved in tryptamine biotransformation, but it appears that there is not a common metabolic pathway, with the nature and position of substituents changing metabolism. Overall, psychedelic tryptamine use is increasing, raising the importance of laboratory identification of specific metabolites to verify intake and identify potential public health NPS outbreaks. The aim of this study was to determine the optimal 4-AcO-DiPT metabolite biomarkers of consumption for clinical and forensic applications, using in silico metabolite prediction, human hepatocyte metabolism, LC-HRMS/MS analysis, and software-assisted data mining.

## 2. Results

### 2.1. In Silico Metabolite Predictions

In silico metabolite predictions are reported in Supplementary Table S1. A total of 47 phase I and II metabolites were predicted, with 10 first-generation metabolites (pM 1–pM 10, by decreasing score) and 37 s-generation metabolites (pM X.1, pM X.4, by decreasing score, and pMX indicating the corresponding first-generation metabolite) (Table 1). First-generation metabolites included hydroxylation, deisopropylation, carboxylation, ester hydrolysis, glucuronidation, and deamination. Second-generation metabolites involved phase I (hydroxylation, dealkylation, carboxylation, and deisopropylation) and phase II (glucuronidation and sulfation) reactions. All predicted metabolites were incorporated in a ddMS^2^ inclusion list to support LC-HRMS/MS analyses (Supplementary Table S1), and all predicted metabolic transformations were incorporated in the list of potential reactions to support automatic data mining.

### 2.2. 4-AcO-DiPT Fragmentation Pattern

**Figure 1:**
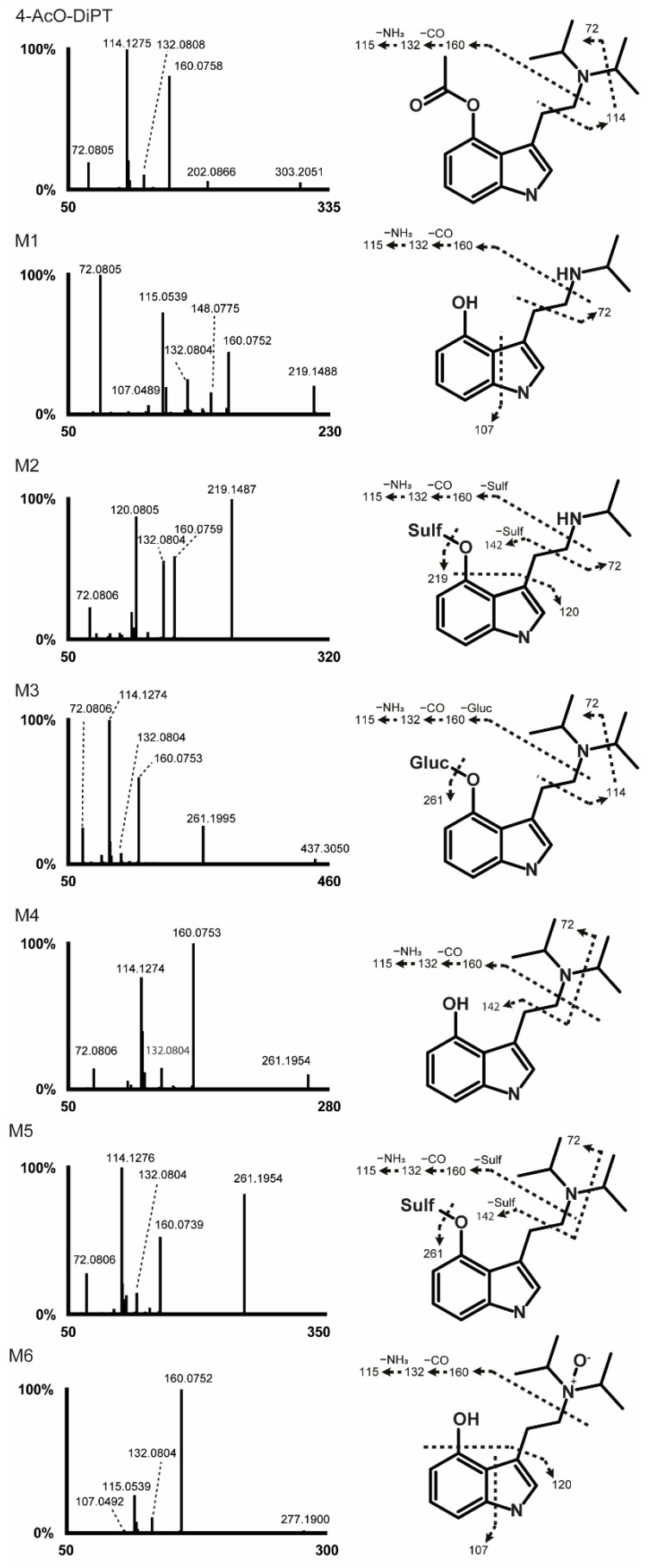
4-AcO-DiPT high-resolution tandem-mass-spectrometry spectrum and suggested fragmentation in positive-ionization mode.

The 4-AcO-DiPT fragmentation pattern is shown in Figure 1. In positive-ionization mode, ions with *m*/*z* 202.0866 resulted from a loss of the diisopropyl amine group followed by loss of the acetyl group producing ions with *m*/*z* 160.0758, the fragment with the most intense signal. Further fragmentation yielded ions with *m*/*z* 132.0808 then 115.0539 through carbon monoxide and ammonia losses, respectively. Ion *m*/*z* 117.0573 was a radical cation typically yielded from hydroxylindole alkylamines [13]. α- and β-cleavage at the nitrogen of the ethylamine chain yielded fragments with *m*/*z* 114.1275 and 102.1278, respectively. Fragments with *m*/*z* 72.0805 were further produced by isopropyl loss from ions with *m*/*z* 114.1275. In addition, the fragments with *m*/*z* 259.1815 in negative ionization mode indicate an acetyl loss on the parent drug.

### 2.3. Metabolite Identification

**Figure 2:**
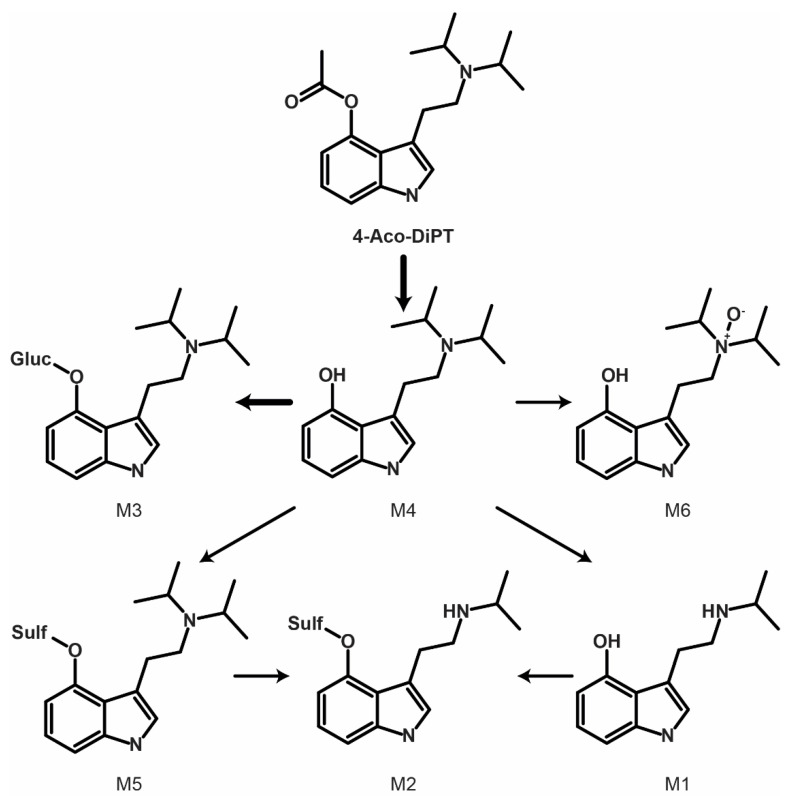
Suggested metabolic fate of 4-AcO-DiPT. Bold indicates major transformations.

**Figure 3:**
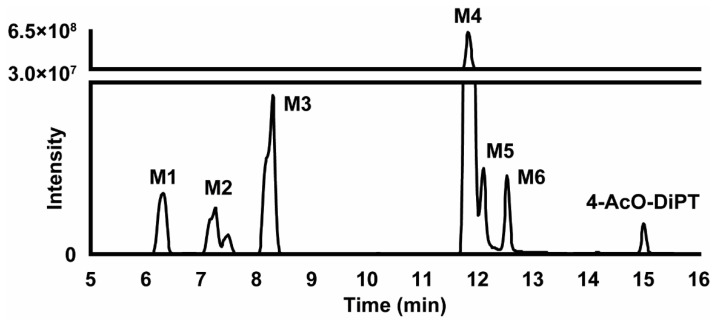
Extracted-ion chromatogram of 4-AcO-DiPT and metabolites in positive-ionization mode obtained after 3 h incubation with human hepatocytes. Mass tolerance: 5 ppm; *m*/*z* values: 219.1489, 261.1955, 277.1907, 299.1057, 303.2059, 341.1525, and 437.2277.

LC-HRMS data were automatically processed to produce a list of six potential metabolites that were manually checked by the operators. The 4-AcO-DiPT LC-HRMS peak area was 3.00 × 10^7^ in the 3 h incubation with hepatocytes, i.e., 375 times lower than that of the 0 h incubation (1.12 × 10^10^). Six metabolites were identified after 3 h incubation following ester hydrolysis, *O*-glucuronidation, *O*-sulfation, *N*-oxidation, and *N*-dealkylation. All second-generation metabolites were derived from the only first-generation metabolite detected, which was produced by ester hydrolysis (4-OH-DiPT, M4). The metabolites are listed from M1 to M6 by ascending retention time (Table 2). The five remaining metabolites all derived from M4 with further phase I and II transformations, including *N*-deisopropylation (4-OH-iPT, M1 and 4-OH-iPT-sulfate, M2) and *N*-oxidation at the ethylamine chain (4-OH-DiPT-*N*-oxide, M6), and *O*-sulfation (M2 and 4-OH-DiPT-sulfate, M5) and *O*-glucuronidation (4-OH-DiPT-glucuronidate, M3) in the indole ring. The fragmentation pattern of 4-AcO-DiPT metabolites is shown in Figure 1. The metabolic pathway of the major metabolites is suggested in Figure 2. An extracted-ion chromatogram of 4-AcO-DiPT and metabolites in positive-ionization mode obtained after 3 h incubation with human hepatocytes is presented in Figure 3.

#### 2.3.1. Phase I Metabolites

##### Ester Hydrolysis

Among the detected metabolites, M4, eluted at 11.82 min, generated the most intense signal with a base peak at *m*/*z* 261.1954. 4-OH-DiPT transformation occurred with the loss of the acetyl group and with hydrolysis occurring in the hydroxyl group of the indole ring, as demonstrated by the production of fragments with *m*/*z* 160.0753, 114.1274, and 117.0573. The fragments from negative-ionization mode at *m*/*z* 132.0243, 114.1275, 132.0804, and 72.0806 showed that the indole core and ethylamine chains were intact. The acetyl loss is indicated by the mass shift of +42.0104 Da from the parent and a difference of -2C -2H -1O on the elemental composition.

M1, M2, M3, M5, and M6 were produced after ester hydrolysis and other metabolic reactions. M*3* was detected as a double peak with the same fragmentation pattern.

##### Ester Hydrolysis and *N*-Deisopropylation

M1 eluted at 6.31 min, and was formed after *N*-deisopropylation following ester hydrolysis. A fragment with *m*/*z* 160.0752 in the MS/MS spectrum clearly identifies that this reaction occurred in the isopropyl group. 4-OH-iPT revealed a mass shift of +84.0570 Da and a difference of elemental composition of -5C -8H–1O proves this metabolic reaction.

##### Ester Hydrolysis and *N*-Oxidation

M6 was detected at 12.53 min and was produced by oxidation on the indole ring after ester hydrolysis. Fragments with *m*/*z* 160.0752, 107.0492, and 115.0539 showed that this transformation did not occur in the indole ring, but instead in the diisopropyl amine group of the molecule. This metabolite presented a mass shift of +26.0152 Da and a difference in elemental composition of -2C -2H. The retention time of 4-OH-DiPT-*N*-oxide was later than that of M4 (4-OH-DiPT), indicating *N*-oxidation [14,15,16].

#### 2.3.2. Phase II Metabolites

##### Ester Hydrolysis and *O*-Glucuronidation

M3, detected at 8.20 min, had the second-most-intense signal. This metabolite was formed after ester hydrolysis and glucuronidation. 4-OH-DiPT-glucuronidate presented a mass shift of −134.0218 Da, differing by +4C +6H +5O from the elemental composition of the parent drug. The presence of fragments with *m*/*z* 160.0753 and 114.1274 clearly indicates no transformation in the indole core or the ethylamine chain. Actually, the signal that was detected in negative ionization mode suggests that a glucuronidation reaction occurred.

##### Ester Hydrolysis and *O*-Sulfation

M5 eluted at 12.11 min, with fragments having *m*/*z* 160.0752 and 115.0539 indicating no reaction at the indole core or the ethylamine chain. 4-OH-DiPT-sulfate showed a mass shift of −37.9466 Da and a difference of -2C -2H +2O +1S on its elemental composition.

##### Ester Hydrolysis, *N*-Deisopropylation, and O-Sulfation

M2, eluting at 7.24 min, resulted from ester hydrolysis, *N*-deisopropylation, and sulfation (produced by M1 *O*-sulfation). The fragments with *m*/*z* 120 prove that no changes were made in the indole core. 4-OH-iPT-sulfate had a mass shift of +4.1002 Da and a difference of -5C -8H +2O +1S in its elemental composition. M2 produced fragments with *m*/*z* 219.1487 by sulfate loss, and similar fragments to those of M1.

The fact that a signal was also identified in negative ionization mode suggests that a sulfation reaction occurred to generate both metabolites (M2 and M5).

## 3. Discussion

In silico metabolite predictions alone are not sufficient to identify the metabolism of specific drugs; however, compilation of HRMS/MS inclusion and exclusion lists and a list of potential metabolic transformations is key to successful Compound Discoverer processing workflow (Supplementary Table S1) and for aiding manual metabolite identification. All six metabolites were predicted. The main phase I and II reactions occurred in the hydroxyl group of the indole ring and in the isopropyl group after ester hydrolysis.

4-AcO-DiPT was incubated with human hepatocytes, which have been proven more suitable for metabolite profiling than human liver microsomes [17]. Hepatocytes are more representative of the physiological liver environment containing both phase I and II drug-metabolizing enzymes, cofactors, and drug transporters [18]. All of these factors allow a better estimation of in vivo metabolism.

The M4 signal was the most intense metabolite signal detected 3 h after incubation with hepatocytes, followed by glucuronidation generating M3. Interestingly, the most intense metabolite (M4) was also detected in the control samples without hepatocytes at 0 and 3 h and at 0 h incubation with hepatocytes but at a much lower intensity than after 3 h incubation, meaning that, although M4 is formed through an enzymatic reaction, it is also spontaneously formed during incubation to a lesser extent. This indicates that M4 formation was overestimated in our experiments. When analyzing authentic samples, digestion (with β-glucuronidase/sulfatase) is preferred to increase the M4 signal. To further understand where the glucuronidation occurred in M3, the 3 h incubations with hepatocytes was reanalyzed after enzymatic hydrolysis with β-glucuronidase, suggesting which part of the molecule was glucuronidated. β-Glucuronidase catalyzes the hydrolysis of *O*-glucuronides and not *N*-glucuronides. Comparing the control sample (same hydrolysis conditions but without β-glucuronidase) to the sample with β-glucuronidase, M3 was not present, revealing that the reaction occurred at the hydroxyl group of the molecule and not on the amine group.

An additional consideration is that samples were resuspended in a ratio of 80:20 mobile phase (A:B) to guarantee maximum recovery of the analyte. This can explain the double peak detected for M3. Better peak shapes would be obtained if samples were resuspended with a lower percentage of acetonitrile (mobile phase B). To assure that the double peak was not an isomer, another test was made using a ratio of 95:5 mobile phase (A:B) where no double peak was detected.

Since the parent drug is most likely degraded as the acetyl group is eliminated quickly, toxicologists could report 4-OH-DiPT (M4)-positive cases as from 4-AcO-DiPT intake. The second-most-intense metabolite was M3 (4-OH-DiPT-glucuronide) followed by M1 (4-OH-iPT), indicating that glucuronidation and dealkylation are common in this tryptamine’s metabolic pathway. Others propose that tryptamines do not have a common metabolic pathway and that metabolism changes depending on the nature and position of their substituents, with demethylation, hydroxylation, and dealkylation being the most common phase I reactions, followed by glucuronidation or sulfation [19,20,21,22,23,24]. 4-OH-DiPT, 4-OH-iPT, and 4-OH-DiPT-*N*-oxide are proposed as biomarkers of 4-AcO-DiPT consumption, but the rapid enzymatic hydrolysis and lower spontaneous hydrolysis of 4-AcO-DiPT to 4-OH-DiPT might create a problem in discerning 4-AcO-DiPT from 4-OH-DiPT consumption. If the parent drug is present even in low concentrations, the ingested drug would be clear, but there is no available information regarding detection of this drug in authentic samples. These results require in vivo confirmation, which is challenging due to the lower prevalence indicated by the small number of 4-AcO-DiPT seizures in recent years.

## 4. Materials and Methods

### 4.1. In Silico Metabolites Prediction

4-AcO-DiPT metabolites were predicted with the online GLORYx freeware [25,26]. The metabolite list was generated using the 4-AcO-DiPT with the “phase I and phase II metabolism” option (Table 1, 1st generation). Metabolites with a score higher than 0.40 were selected and reprocessed to simulate a second-step metabolism reaction (Table 1, 2nd generation); the second-generation metabolite score was multiplied by the first-generation metabolite score and scores higher than 0.20 were added to the inclusion list (Supplementary Table S1).

### 4.2. Chemicals and Reagents

4-AcO-DiPT was obtained from Cayman Chemical (Ann Arbor, MI, USA) and diclofenac was acquired from Sigma Aldrich (Milan, Italy). Stock standards of these chemicals were prepared to 1 mg/mL in LC-MS-grade methanol (Carlo Erba; Cornaredo, Italy). Standards were stored at –20 °C until analysis. Ten-donor-pooled cryopreserved human hepatocytes, thawing medium (TM), and 0.4% trypan blue were purchased from Lonza (Basel, Switzerland). l-Glutamine, HEPES (2-[4-(2-hydroxyethyl)-1-piperazinyl]ethane-sulfonic acid), and Williams’ Medium E were obtained from Sigma Aldrich. l-Glutamine and HEPES were dissolved in Williams’ Medium E to 2 and 20 mmol/L, respectively, prior to analysis. The supplemented Williams’ Medium E (sWME) was stored at 4 °C until incubation. LC-MS-grade acetonitrile, water, and formic acid were obtained from Carlo Erba (Cornaredo, Italy).

### 4.3. Hepatocytes Incubation

Incubations were conducted as previously described, with small adjustments [27]. Hepatocytes, thawed at 37 °C, were lightly mixed with 50 mL TM in a 50 mL polypropylene conical tube maintained at the same temperature. The tube was centrifuged at 100× *g* for 5 min and the pellet washed with 50 mL sWME at 37 °C. After centrifugation under the same conditions, cells were resuspended in 2 mL sWME. Hepatocyte viability was evaluated with the trypan blue exclusion test, and sWME volume adjusted to 2 × 10^6^ viable cells/mL. Incubations were prepared in sterile 24-well culture plates with 250 μL hepatocyte suspension and 250 μL 4-AcO-DiPT at 20 μmol/L in sWME at 37 °C. Metabolic reactions were then interrupted with 500 µL ice-cold acetonitrile. Negative controls, i.e., hepatocytes in sWME without 4-AcO-DiPT and 4-AcO-DiPT in sWME without hepatocytes, were incubated for 3 h under the same conditions. Diclofenac was also incubated under the same conditions to ensure proper metabolic activity.

### 4.4. Sample Preparation

For sample preparation, 100 μL hepatocyte supernate was vortexed with 100 μL acetonitrile, centrifuged at 15,000× *g* for 10 min at room temperature, and the supernates were dried under nitrogen at 37 °C. Residues were reconstituted with 150 μL mobile phase A (MPA): mobile phase B (MPB) (8:2 *v*/*v*), mixed well, and centrifuged for 10 min at 15,000× *g* at room temperature, and the supernates were transferred into glass inserts in LC autosampler vials. The sample injection volume was 15 μL.

### 4.5. Instrumental Conditions

LC-HRMS/MS analyses were performed on a DIONEX UltiMate 3000 liquid chromatograph coupled with a Q-Exactive quadrupole-Orbitrap hybrid high-resolution mass spectrometer with a heated-electrospray-ionization (HESI) source (Thermo Scientific, Waltham, MA, USA).

### 4.6. Liquid Chromatography

Chromatographic separation occurred on a Kinetex^®^ Biphenyl column (150 × 2.1 mm, 2 μm) (Phenomenex, Castel Maggiore, Italy) maintained at 37 ± 1 °C. The 30 min run utilized 0.1% formic acid in water (MPA) and 0.1% formic acid in acetonitrile (MPB) at a 0.4 mL/min flow rate. The gradient was started at 5% MPB for 2 min, increased to 25% MPB by 18 min, further increased to 95% MPB within 2 min, and held for 5 min. The return to initial conditions occurred within 0.1 min, followed by a 4.9 min equilibration, for a total runtime of 30 min. Autosampler temperature was 10 ± 1 °C.

### 4.7. Mass Spectrometry Conditions

Samples were injected twice, once in positive- and once in negative-ionization mode. HESI source parameters were 50 sheath-gas flow rate, 10 auxiliary-gas flow rate, ±3 kV spray voltage, 300 °C capillary- and auxiliary-gas heater temperature, and 50 S-lens radio frequency; sweep gas was not applied. The orbitrap was calibrated prior to analysis, with a lock mass list for better accuracy. Data were acquired from 1 to 25 min in full-scan HRMS (FullMS)/data-dependent MS/MS (ddMS^2^) mode. The FullMS acquisition range was *m*/*z* 120–700 with a resolution of 70,000 at full width at half maximum (FWHM) at *m*/*z* 200. The automatic gain control (AGC) target was 10^6^ and maximum injection time (IT) 200 ms. Up to 5 ddMS^2^ scans were triggered, with a dynamic exclusion of 2.0 s and an intensity threshold of 10^4^ for each FullMS scan, depending on a priority inclusion list of putative metabolites (Supplementary Table S1) based on in silico predictions and the metabolic fate of 4-AcO-DiPT analogues (Table 1). Other ions not in the inclusion list might also trigger ddMS^2^ scans. Additionally, background *m*/*z* values with high intensity were assessed during injection of blank controls and compiled in an exclusion list in positive- and negative-ion modes. The ddMS^2^ isolation window was *m*/*z* ± 1.2 with a resolution of 17,500 and the normalized collision energy (NCE) was 30, 60, and 90 a.u. The ddMS^2^ AGC target was 2 × 10^5^ and maximum IT was 64 ms.

### 4.8. Final Metabolite Identification

LC-HRMS data were processed with Compound Discoverer (v. 3.1.1.12) from Thermo Scientific (Waltham, MA, USA), using a partially automated approach. Briefly, the ions detected in HRMS were compared to a list of theoretical metabolites (intensity threshold, 5 × 10^3^; mass tolerance, 5 ppm). The HRMS/MS spectra and theoretical elemental composition of the ions were compared to online databases (intensity threshold, 10^5^; HRMS mass tolerance, 5 ppm; HRMS/MS mass tolerance, 10 ppm). The chromatographic peaks detected in controls with a similar or higher intensity to that of the peaks detected in the samples were filtered out.

## 5. Conclusions

This study marks the first characterization of the 4-AcO-DiPT metabolic profile in human hepatocytes using in silico metabolite predictions, LC-HRMS/MS analysis, and software-assisted data mining. We identified six metabolites through ester hydrolysis followed by *N*-Deisopropylation, *O*-sulfation, *O*-glucuronidation, and/or *N*-Oxidation. We recommend 4-OH-DiPT, 4-OH-iPT, and 4-OH-DiPT-*N*-oxide as metabolite biomarkers of 4-AcO-DiPT consumption in clinical and forensic toxicology. We propose incorporation of HRMS/MS fragmentation patterns into the online libraries mzCloud and HighResNPS. These findings underscore the value of experimental data and demonstrate how challenging it is to predict NPS metabolism. Our in vitro model offers preliminary findings about 4-AcO-DiPT human metabolism; however, these findings should be verified using samples from authentic positive cases. These data enable analytical toxicologists to identify 4-AcO-DiPT exposure cases.

## Figures and Tables

**Table 1:** Molecular structure, elemental composition, metabolic transformation, and predictive score of in silico-predicted 4-AcO-DiPT metabolites.

Predicted Metabolite (pM)		Transformation	Elemental Composition	Score (%)
pM1		*N*-Hydroxylation	C_18_H_27_N_2_O_3_	63
	pM1.1	*O*-Glucuronidation	C_25_H_30_N_2_O_8_	30
	pM1.2	*N*-Deisopropylation	C_16_H_21_NO_3_	22
	pM1.3	Hydroxylation	C_19_H_28_NO_4_	22
	pM1.4	Hydroxylation (C1″)	C_18_H_26_N0_4_	21
pM2		Hydroxylation (C2″)	C_18_H_26_N_2_O_3_	63
	pM2.1	*O*-Sulfation	C_18_H_24_N_3_O_7_S	60
	pM2.2	*O*-Glucuronidation	C_24_H_32_N_2_O_10_	39
	pM2.3	Dealkylation	C_17_H_24_N_2_O_2_	21
	pM2.4	Hydroxylation (C11)	C_19_H_26_N_2_O_4_	21
	pM2.5	Carboxylation (C9)	C_19_H_24_N_2_O_5_	21
pM3		Deisopropylation	C_15_H_20_N_2_O_2_	63
	pM3.1	Hydroxylation (C1″)	C_16_H_20_N_2_O_3_	35
	pM3.2	Depropylation	C_13_H_14_O_3_	35
	pM3.3	*N*-Hydroxylation	C_15_H_20_N_2_O_3_	35
	pM3.4	*N*-Deisopropylation	C_12_H_14_N_2_O_2_	35
pM4		Hydroxylation (acetyl)	C_18_H_26_N_2_O_3_	34
	pM4.1	*O*-Glucuronidation	C_23_H_32_N_2_0_9_	28
	pM4.2	*O*-Sulfation	C_17_H_24_N_2_0_6_S	24
pM5		Carboxylation (acetyl)	C_19_H_26_N_2_O_3_	34
	pM5.1	*O*-Glucuronidation	C_26_H_36_N_2_O_9_	31
	pM5.2	*N*-Hydroxylation	C_18_H_25_N_2_O_5_	20
	pM5.3	Deisopropylation	C_15_H_18_N_2_0_4_	20
	pM5.4	Hydroxylation (C1″)	C_18_H_24_N_2_O_5_	20
pM6		Ester hydrolysis	C_19_H_26_N_2_O	34
	pM6.1	*O*-Sulfation	C_16_H_24_N_2_O_4_S	32
	pM6.2	*O*-Glucuronidation	C_22_H_32_N_2_O_7_	29
	pM6.3	Hydroxylation (C1″)	C_16_H_24_N_2_O_2_	20
	pM6.4	*N*-Hydroxylation	C_16_H_25_N_2_O_2_	20
pM7		Hydroxylation (C2)	C_16_H_24_N_2_O_2_	25
pM8		*N*-Glucuronidation	C_12_H_13_NO_3_	20
pM9		Deamination (to aldehyde)	C_12_H_13_NO_3_	20
pM10		Deamination (to alcohol)	C_12_H_11_NO_3_	20

**Table 2:** Metabolic transformation, retention time (RT), accurate mass of molecular ion (hydrogen adduct in positive-ionization mode [M + H]^+^), elemental composition, deviation from theoretical accurate mass, and liquid chromatography–high-resolution mass spectrometry peak area of 4-AcO-DiPT and metabolites after 3 h incubation with human hepatocytes (HESI positive mode, HESI negative mode).

ID	Transformation	RT (min)	[M + H]^+^	Elemental Composition	Mass Error (ppm)	Peak Areas (HESI+, HESI−)
M1	Ester hydrolysis + *N*-Deisopropylation	6.31	219.1489	C_13_H_18_N_2_O	−1.21	1.10 × 10^8^
M2	Ester hydrolysis + *N*-Deisopropylation + *O*-Sulfation	7.24	299.1057	C_13_H_18_N_2_O_4_S	−1.23	3.50 × 10^7^, 5.90 × 10^7^
M3	Ester hydrolysis + *O*-Glucuronidation	8.30	437.2277	C_22_H_32_N_2_O_7_	−1.45	2.94 × 10^8^, 2.60 × 10^7^
M4	Ester hydrolysis	11.82	261.1955	C_16_H_24_N_2_O	−2.25	4.19 × 10^9^
M5	Ester hydrolysis + *O*-Sulfation	12.11	341.1525	C_16_H_24_N_2_O_4_S	−1.42	4.47 × 10^7^, 2.79 × 10^7^
M6	Ester hydrolysis + *N*-Oxidation	12.53	277.1907	C_16_H_24_N_2_O_2_	−1.35	7.76 × 10^6^
Parent	no transformation	15.02	303.2059	C_18_H_26_N_2_O_2_	−2.67	3.00 × 10^7^

## Data Availability

The data presented in this study are available in the main article and the supplementary materials.

## Supplementary Materials

The following supporting information can be downloaded at: https://www.mdpi.com/article/10.3390/metabo12080705/s1, Table S1: Inclusion list for the MS/MS data-dependent acquisition.
