# Supplementary material for: Human Hepatocyte 4-Acetoxy-N,N-Diisopropyltryptamine Metabolite Profiling by Reversed-Phase Liquid Chromatography Coupled with High-Resolution Tandem Mass Spectrometry
Source: Metabolites. 2022 Jul 29;12(8):705. doi: 10.3390/metabo12080705 (PMC9413566; doi:10.3390/metabo12080705)
Supplement: Supplementary file 1 [file metabolites-12-00705-s001.zip › metabolites-1821677-supplementary.pdf]

Supplementary Table S1. Inclusion list for the MS/MS data-dependent acquisition.

Transformation	Molecular formula	$[M + H]^+$ (m/z)	$[M - H]^-$ (m/z)
Parent (4-AcO-DiPT)	$C_{18}H_{26}N_2O_2$	303.2067	301.1922
-2C-2H-O	$C_{16}H_{24}N_2O$	261.1961	259.1816
+O	$C_{18}H_{26}N_2O_3$	319.2016	317.1871
-3C-6H	$C_{15}H_{20}N_2O_2$	261.1598	259.1452
+O-2H	$C_{18}H_{24}N_2O_3$	317.1860	315.1714
+4C+6H+5O	$C_{22}H_{32}N_2O_7$	437.2282	435.2137
-2C-2H+2O+S	$C_{16}H_{24}N_2O_4S$	341.1530	339.1384
-2C-2H+2O+S	$C_{16}H_{24}N_2O_4S$	341.1530	339.1384
+2H+2O	$C_{18}H_{28}N_2O_4$	337.2122	335.1976
-6C-13H-N+O	$C_{12}H_{13}NO_3$	220.0968	218.0823
-6C-15H-N+O	$C_{12}H_{11}NO_3$	218.0812	216.0666
+10C+17H+3N+7O+S	$C_{28}H_{43}N_5O_9S$	626.2854	624.2709
-6C-15H-N+2O	$C_{12}H_{11}NO_4$	234.0761	232.0615
-2C-2H	$C_{16}H_{24}N_2O_2$	277.1911	275.1765
-5C-8H-O	$C_{13}H_{18}N_2O$	219.1492	217.1346
+2O	$C_{18}H_{26}N_2O_4$	335.1965	333.1820
+6C+8H+7O	$C_{24}H_{34}N_2O_9$	495.2337	493.2192
+4O+S	$C_{18}H_{26}N_2O_6S$	399.1584	397.1439
-3C-6H+O	$C_{15}H_{20}N_2O_3$	277.1547	275.1401
-6C-12H	$C_{12}H_{14}N_2O_2$	219.1128	217.0983
+4C+6H+6O	$C_{22}H_{32}N_2O_8$	453.2231	451.2086
-2C-2H+3O+S	$C_{16}H_{24}N_2O_5S$	357.1479	355.1333
-5C-8H	$C_{13}H_{18}N_2O_2$	235.1441	233.1296
-2C-2H+O	$C_{16}H_{24}N_2O_3$	293.1860	291.1714
-2C-4H	$C_{16}H_{22}N_2O_2$	275.1754	273.1609
-8C-17H-N	$C_{10}H_9NO_2$	176.0706	174.0561
-8C-17H-N+O	$C_{10}H_9NO_3$	192.0655	190.0510
-3C-8H+O	$C_{15}H_{18}N_2O_3$	275.1390	273.1245
-2C+O	$C_{16}H_{26}N_2O_3$	295.2016	293.1871
+8C+15H+3N+6O+S	$C_{26}H_{41}N_5O_8S$	584.2749	582.2603
+C+6O	$C_{19}H_{26}N_2O_8$	411.1762	409.1616
-5C-8H+3O+S	$C_{13}H_{18}N_2O_5S$	315.1009	313.0864
+4C+6H+7O	$C_{22}H_{32}N_2O_9$	469.2181	467.2035
-2C-2H+4O+S	$C_{16}H_{24}N_2O_6S$	373.1428	371.1282
+C+5O	$C_{19}H_{26}N_2O_7$	395.1813	393.1667
-8C-14H-O	$C_{10}H_{12}N_2O$	177.1022	175.0877
-5C-10H	$C_{13}H_{16}N_2O_2$	233.1285	231.1139
-8C-16H	$C_{10}H_{10}N_2O_2$	191.0815	189.0670
-5C-8H+2O+S	$C_{13}H_{18}N_2O_4S$	299.1060	297.0915

-2C-4H+O	$C_{16}H_{22}N_2O_3$	291.1703	289.1558
+6C+8H+8O	$C_{24}H_{34}N_2O_{10}$	511.2286	509.2141
+5O+S	$C_{18}H_{26}N_2O_7S$	415.1534	413.1388
-6C-14H+O	$C_{12}H_{12}N_2O_3$	233.0921	231.0775
-2H+2O	$C_{18}H_{24}N_2O_4$	333.1809	331.1663
-C-O	$C_{17}H_{26}N_2O$	275.2118	273.1972
+C+2H+O	$C_{19}H_{28}N_2O_3$	333.2173	331.2027
-C	$C_{17}H_{26}N_2O_2$	291.2067	289.1922
-2H	$C_{18}H_{24}N_2O_2$	301.1911	299.1765
-2C-4H-O	$C_{16}H_{22}N_2O$	259.1805	257.1659
